# Transcriptome analysis of fasudil treatment in the APPswe/PSEN1dE9 transgenic (APP/PS1) mice model of Alzheimer’s disease

**DOI:** 10.1038/s41598-022-10554-9

**Published:** 2022-04-22

**Authors:** Hailong Yan, Yuqing Yan, Ye Gao, Nianping Zhang, Gajendra Kumar, Qingli Fang, Ziqing Li, Jiehui Li, Yuna Zhang, Lijuan Song, Jiawei Wang, Jingxian Sun, Han-Ting Zhang, Cun-Gen Ma

**Affiliations:** 1 Institute of Brain Science, Shanxi Key Laboratory of Inflammatory Neurodegenerative Diseases, Medical School of Shanxi Datong University, Datong, 037009 China; 2 The Key Research Laboratory of Benefiting Qi for Acting Blood Circulation, Research Center of Neurobiology, Shanxi University of Chinese Medicine, Jinzhong, 030619 China; 3 Department of Neuroscience, City University of Hong Kong, Tat Chee Avenue, Kowloon Tong, Hong Kong; 4 Department of Pharmacology, Qingdao University School of Pharmacy, Qingdao, 266073 China

**Keywords:** Molecular biology, Neuroscience

## Abstract

Alzheimer's disease (AD) is the most common cause of progressive dementia. In the present study, we showed hippocampal tissue transcriptome analysis in APPswe/PSEN1dE9 (APP/PS1, AD model) mice treated with fasudil (ADF) and compared with AD mice treated with saline (ADNS) and wild type mice (WT). The competing endogenous RNA (ceRNA) network was constructed and validated the differential expression of mRNA, lncRNA, miRNA, and circRNA. Our study showed differentially expressed mRNAs (DEMs) between WT and ADNS, while enriched in cell growth and death and nervous system pathways. DEMs between ADNS-ADF were enriched in the nervous system, glycosaminoglycan biosynthesis-keratan sulfate (KS) and Quorum sensing pathways. We validated four genes with RT-PCR, whereas enrichment of Acyl-CoA Synthetase Long Chain Family Member 4 (Acsl4, ENSMUST00000112903) in Quorum sensing pathways, and BTG anti-proliferation factor 1 (Btg1, ENSMUST00000038377) in RNA degradation pathways were conducted. Expression of these two genes were higher in ADNS, but were significantly reduced in ADF. Histone H4 transcription factor (Hinfp, ENSMUST00000216508) orchestrate G1/S transition of mitotic cell cycle and co-expressed with mmu-miR-26a-2-3p-mediated ceRNA and mmu-miR-3065-5p-mediated ceRNA; Wnt family member 4 (Wnt4, ENSMUST00000045747) was enriched in mTOR, Hippo and Wnt signaling pathway. Expression of these two genes were significantly lower in ADNS, and fasudil treatment reverse it. The present studies demonstrated four genes: Acsl4, Btg1, Hinfp, Wnt4 could be potential biomarkers of AD and the targets of fasudil treatment. These results will pave a novel direction for future clinic studies for AD and fasudil treatment.

## Introduction

Alzheimer’s disease (AD) is the most common neurodegenerative disease and one of the major sources of morbidity and mortality in the aging population^1^, and the etiology of which is multifaceted and poorly understood. It is urgently required to develop effective treatments for AD^2^. In recent years, noncoding RNAs (ncRNAs), including long non-coding RNAs (lncRNAs), circular RNAs (circRNAs), and microRNAs (miRNAs), have emerged as a major class of regulatory molecules, which play a significant role in the pathogenesis of AD^3,4^. The ncRNAs detected in the cerebrospinal fluid (CSF) have been reported as potential biomarkers for symptomatic and course-altering therapy^5–7^, and are involved in multiple pathways associated with disease condition. However, it is important to pay attention and dissect the possible ncRNA-induced pathogenesis and drug targets to control the devastating dementia of AD in the future^8,9^.

Long non-coding RNAs (lncRNAs) consist of > 200 nt regulating protein transcription and epigenetic modification in several diseases^10–12^. LncRNA was initially considered as the "noise" of genome transcription, a by-product of RNA polymerase II transcription with no biological function^13^. However, studies have shown that lncRNA is involved as important regulators such as X chromosome silencing, genomic imprinting, chromatin modification, transcriptional activation, transcriptional interference, and nuclear transport^14–17^. For example, lncRNA n336694 and miR-106b are over expressed in brain tissues of AD mice^18^. LncRNA MALAT1 appears to interact with miR-125b to inhibit neuron apoptosis and inflammation while promotes neurite outgrowth in AD^19,20^. lncRNA ANRIL knockdown suppresses apoptosis and inflammation and enhances neurite extension via binding to microRNA-125a in the PC12 cellular model of AD^21^.

miRNAs (around 22 nt in length) are typically associated with the 3′-untranslated region (UTR) of targeted mRNAs and repress their translation and/or stability^22^ and also important regulators of gene expression at the post-transcriptional level; the levels of miRNA let-7 are enhanced in AD patients^23,24^.

circRNAs are derivatives of precursor mRNAs (pre-mRNAs), and formed covalently by linked 5′ and 3′ ends called “back-splicing”, also known as “head to tail junction”^25,26^, which are abundant in the brain and play an important role in synaptopathologies. Neural circRNAs accumulate with age and have a beneficial role in neuronal repair^27,28^. Recently, it has been reported in a mouse model of AD that circRNA dysfunction (94 and 141 circRNAs) dysregulates 3 circRNAs involved in amyloid-β plaque clearance^26^.

Rho-associated protein kinase (ROCK) inhibitors are a series of compounds that target rho kinase. Rho kinase inhibitor fasudil, a small molecule targeting ROCK II, which has been reported by our previous studies as a treatment of AD, but none of the studies have analyzed the transcriptome after fasudil treatment in AD.

In the present study, we analyzed the diverse RNAs altered after fasudil treatment in AD mice model and investigated the function of those RNAs. We expected that our observation and functional analysis of the transcriptome in AD will be an important finding for determining the neurological interaction in AD pathogenesis.

## Results

### Fasudil treatment altered the expression of Aβ and p-Tau in hippocampus of APPswe/PSEN1dE9 mice

**Figure 1:**
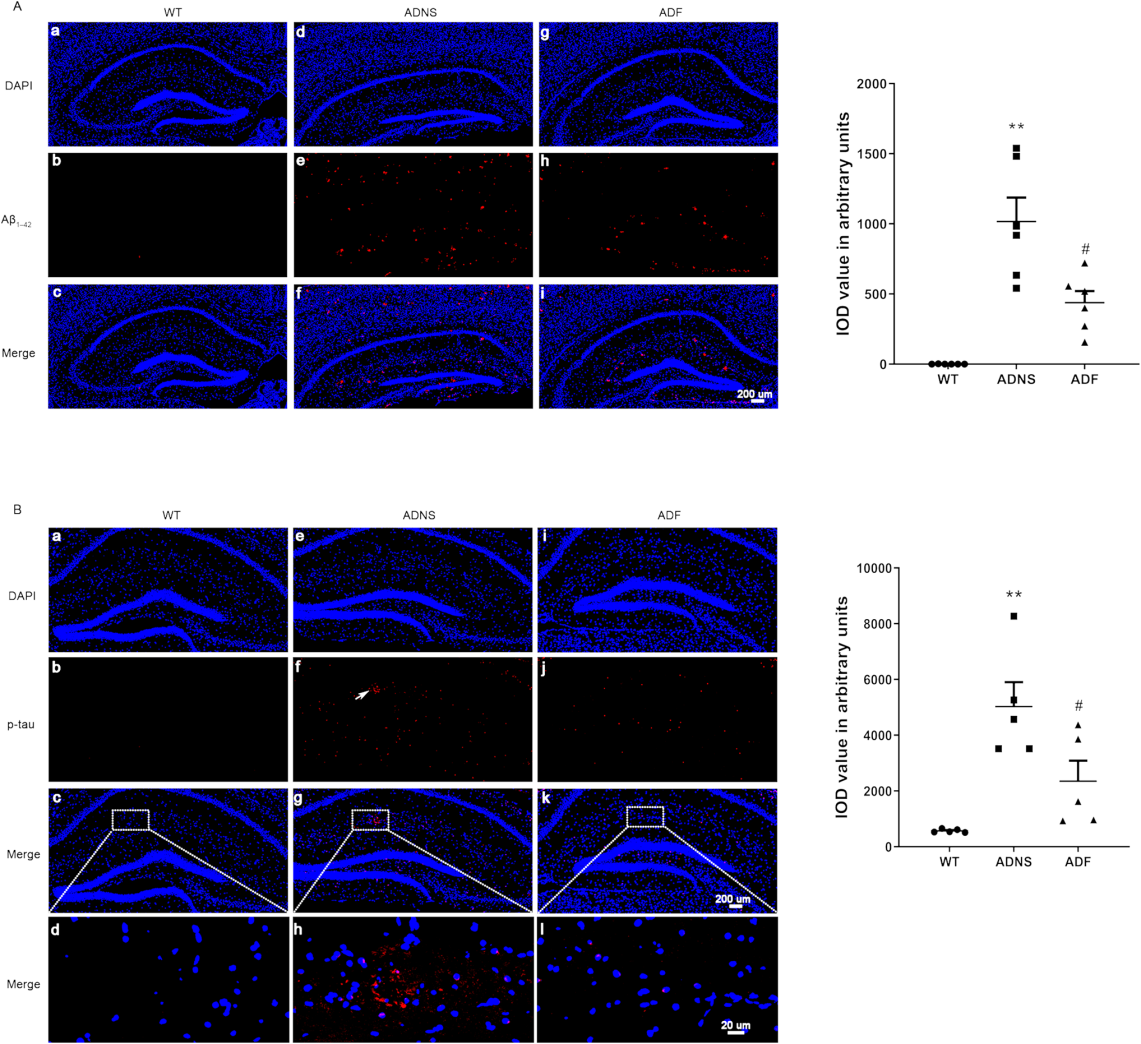
Fasudil treatment reduced Aβ plaque deposition and phosphorylated tau tangles in the hippocampus. (**A**) Aβ_1-42_ plaques was checked in the hippocampus of the mice in each group. (a–c) WT, (d–f) ADNS, (g–i) ADF. Immunofluorescent intensity quantification of anti-Aβ_1-42_ showed significant increased intensity in ADNS group when compared with WT (*p* < 0.01) and the reverse in the Fasudil-treated mice, suggesting the protective effect on Aβ burden and therapeutic potential for clearance of Aβ plaques in AD with Fasudil treatment. **(B)** Tau phosphorylation at S404 was checked in the hippocampus of the mice in each group. (a–d) WT group, (e–h) ADNS group, (i–l) ADF group. Images (a–c, e–h, i–l) were taken at 10× (200 μm). The box area is enlarged, and the images (d, h, l) were taken at 60× (20 μm). DAPI (blue) was used for nuclei staining. Immunofluorescent intensity quantification of anti-Phospho-Tau (S404) showed a significant increase in intensity in the ADNS group when compared with WT (*p* < 0.01); the reverse was observed in the Fasudil-treated mice. Data is presented as Mean ± S.E.M. *p < 0.05, **p < 0.01 versus WT; #*p* < 0.05, ##*p* < 0.01 versus ADNS. The Dunnett’s test were used for statistical analysis.

Aβ and p-Tau are biomarkers of Alzheimer’s disease^29,30^. Expression of Aβ and p-Tau in the hippocampus of WT, ADNS, and ADF mice were evaluated by immunostaining. Our results showed that ADNS induced Aβ_1-42_ plaques (1016.59) in the hippocampus; these were significantly reduced in ADF (437.8; *p* < 0.01), suggesting that fasudil reduced Aβ burden (*p* < 0.05) (Fig. 1A). Tau phosphorylation is a key factor playing an important role in the pathogenicity of AD^31^. The expression of p-Tau (S404) in hippocampal neurons of ADNS was significantly increased as compared to WT (5029.2) (*p* < 0.01). However, it was reversed by fasudil (2353.7) as shown by immunofluorescent intensity quantification (*p* < 0.05), suggesting the protective effect of fasudil in terms of decreasing p-Tau formation in AD (Fig. 1B).

### Transcriptome Analysis of hippocampal tissues of APPswe/PSEN1dE9 mice treated with fasudil

Protein-coding (mRNAs) and noncoding RNAs (lncRNAs, miRNAs, circRNAs) associated with fasudil treatment in APPswe/PSEN1dE9 mice were analyzed using samples of hippocampal tissues from WT, ADF and ADNS (n = 3 in each group), as described below.

### Analysis of differentially expressed protein-coding transcripts (mRNA) in fasudil-treated APPswe/PSEN1dE9 mice

**Figure 2:**
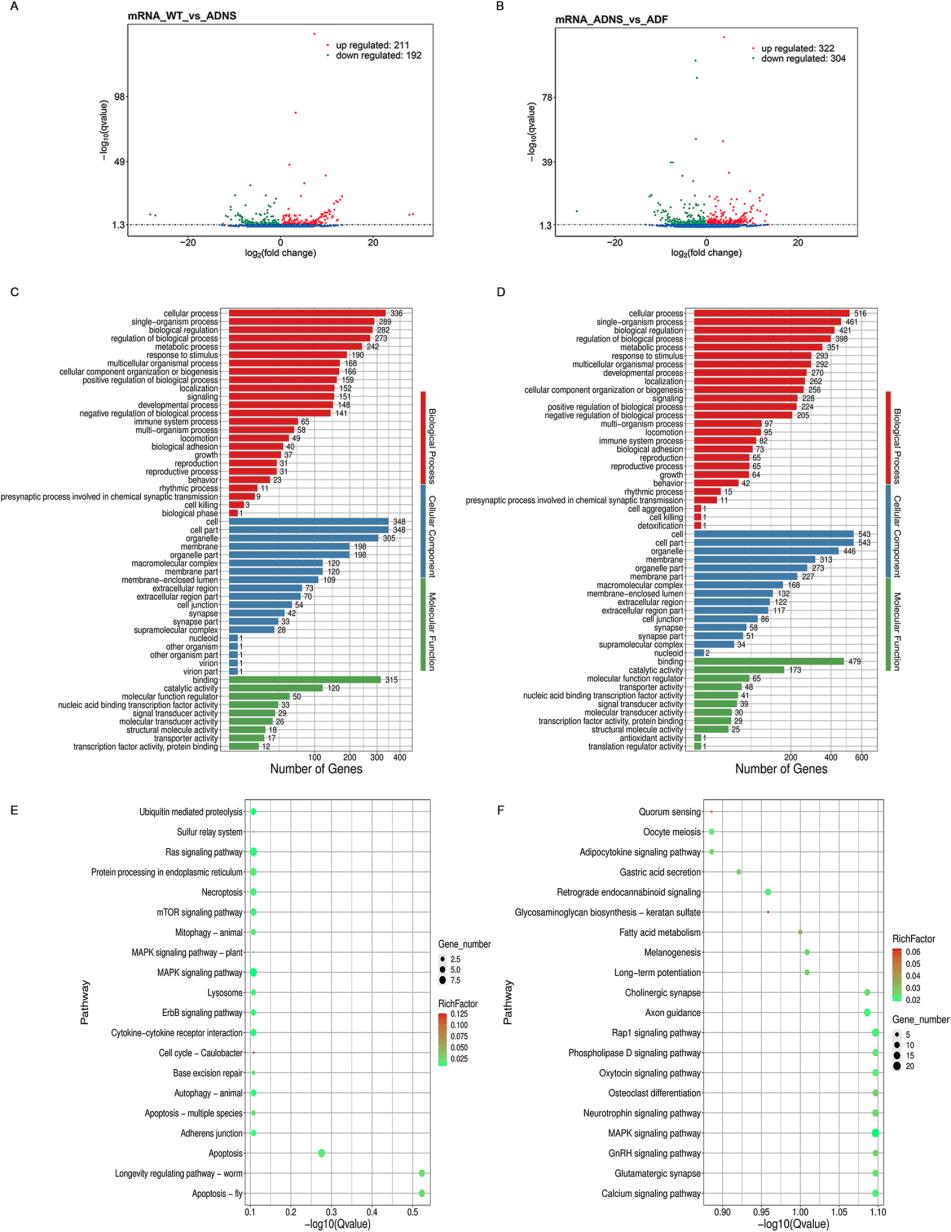
Differentially expressed mRNAs in the hippocampal tissues from mice in each group. (**A**) The number of upregulated and downregulated mRNAs between WT and ADNS are shown; 403 (211 up and 192 down) differentially expressed mRNAs (DEMs) between WT versus ADNS. **(B)** The number of upregulated and downregulated DEMs between ADF and ADNS are shown; 626 (322 up and 304 down) DEMs were identified between ADNS versus ADF. **(C)** GO annotations of 403 DEMs in WT versus ADNS. The mRNAs were significantly enriched in 1103 GO terms (*p* < 0.05). **(D)** GO analysis of 626 DEMs in ADNS versus ADF. The mRNAs were significantly enriched in 1199 GO terms (*p* < 0.05). **(E)** KEGG pathways involving the top 20 terms in WT versus ADNS. A larger Rich Factor value indicates a higher degree of enrichment. DEMs between WT-ADNS were enriched from cell growth and death pathways including the cell cycle and apoptosis pathway. **(F)** KEGG pathways involving the top 20 terms in ADNS versus ADF. DEMs between ADNS-ADF were enriched from nervous system pathways including neurotrophin signaling pathway, glutamatergic synapse, cholinergic synapse, long-term potentiation and retrograde endocannabinoid signalling (KEGG pathways: https://www.kegg.jp/kegg/kegg1.html).

We obtained on average 17.17G of clean data for each library. The sequencing quality showed > 97.5% Q20 and > 93.1% Q30 (Supplementary Table 1). Out of all the sequencing libraries, we identified 65,151 protein-coding transcripts, including 57,726 known and 7,425 novel protein-coding transcripts. Overall, this resulted in 403 differentially expressed mRNAs (DEMs) between WT versus ADNS, including 192 down-regulated (47.7%) and 211 up-regulated (52.4%), and 626 DEMs between ADNS versus ADF, including 304 down-regulated (48.6%) and 322 up-regulated (51.4%) (Fig. 2A,B; Supplementary Table 2).

Further, we identified the biological functions of the DEMs between WT versus ADNS and ADNS versus ADF by gene ontology (GO) analysis. In both WT versus ADNS and ADNS versus ADF, our results showed that the most important molecular functions are binding and catalytic activity. In biological processes, the dominant GO terms were cellular process, single organism process, and biological regulation. The key cellular components involved were cell, cell part, organelle and membrane (Fig. 2C,D; Supplementary Table 3).

### Analysis of differentially expressed protein-coding transcripts (mRNA) in fasudil-treated APPswe/PSEN1dE9 mice

In the KEGG pathway analysis, DEMs between WT-ADNS were enriched in the cell growth and death pathways including the cell cycle and apoptosis pathway; 11 genes were associated with the above pathways (Fig. 2E; Supplement Table 4). DEMs between ADNS-ADF were enriched in the nervous system pathways including the neurotrophin signaling pathway, glutamatergic synapse, cholinergic synapse, long-term potentiation, retrograde endocannabinoid signaling, long-term depression, dopaminergic synapse, glycosaminoglycan biosynthesis-keratan sulfate (KS) pathways. A total of 35 genes were associated with the above pathways. DEMs between ADNS-ADF were enriched in Glycosaminoglycan biosynthesis—keratan sulfate pathways and Quorum sensing pathways (Fig. 2F; Supplementary Table 4). Acyl-CoA Synthetase Long Chain Family Member 4 (Acsl4, ENSMUST00000112903) were enriched in Quorum sensing pathways. BTG anti-proliferation factor 1 (Btg1, ENSMUST00000038377) was enriched in RNA degradation pathways and significantly reduced in ADF. Wnt family member 4 (Wnt4, ENSMUST00000045747) was enriched in signal transduction pathways, including mTOR, Hippo and Wnt signaling pathway (Supplementary Table 4).

### Analysis of lncRNA changes in fasudil-treated APPswe/PSEN1dE9 mice

**Figure 3:**
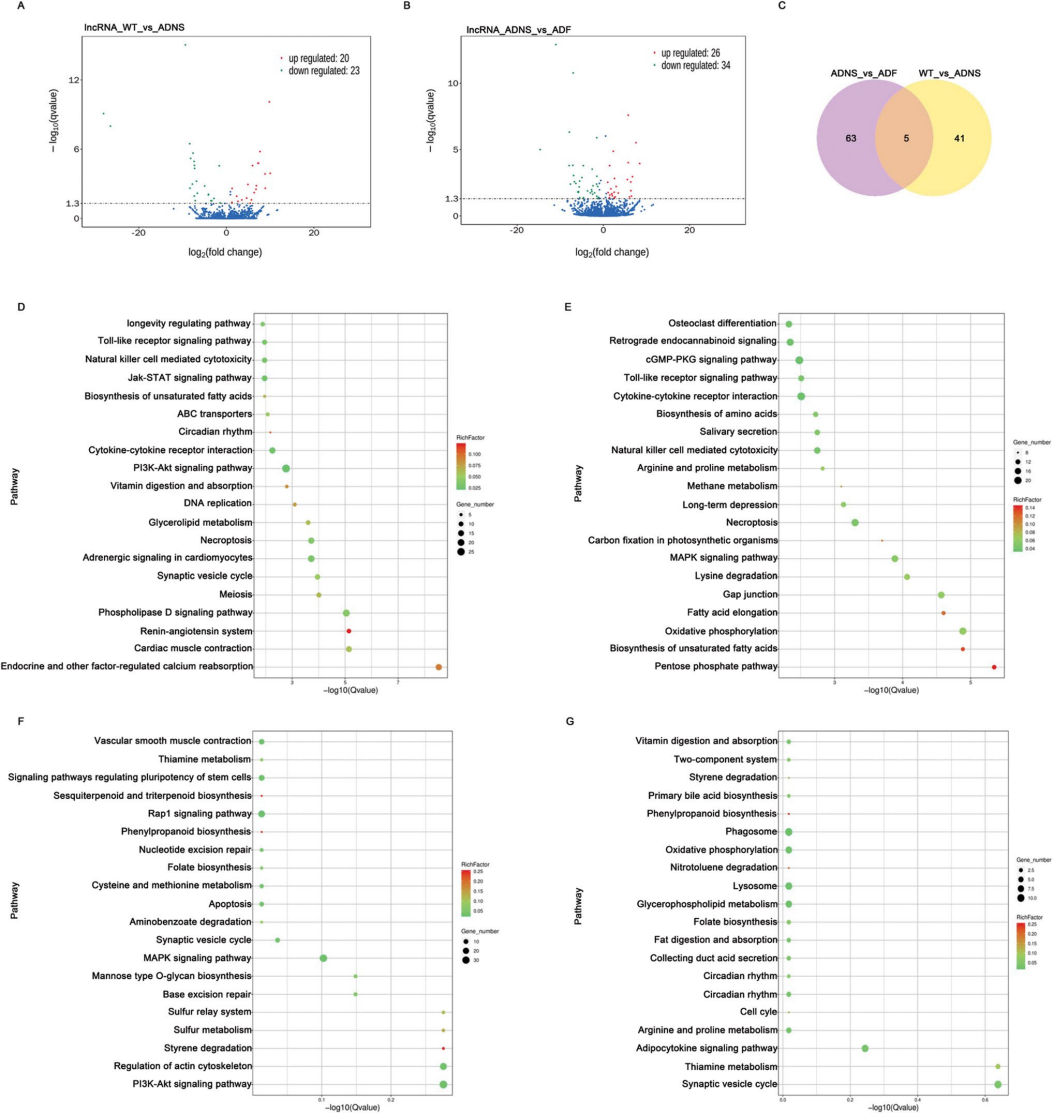
Differentially expressed lncRNAs in hippocampal tissues from mice in each group. (**A**) The number of upregulated (22) and downregulated (24) lncRNAs between ADNS and WT are shown. **(B)** The number of upregulated (29) and downregulated (39) differentially expressed lncRNAs between ADF and ADNS are shown. **(C)** Venn showed 5 lncRNAs at the intersection of ADNS versus ADF and WT versus ADNS. **(D)** KEGG analysis involving the top 20 terms of differentially expressed lncRNA co-located with mRNA in WT versus ADNS. Most genes focus in PI3K-AKT signal pathway. **(E)** KEGG analysis involving the top 20 terms of differentially expressed lncRNA co-located with mRNA in ADNS versus ADF. Most genes focus in cytokine-cytokine receptor interaction and cGMP-PKG signaling pathway. **(F)** KEGG analysis of differentially expressed lncRNA co-expressed with mRNA in WT versus ADNS. Most genes focus on the pathway of PI3K-Akt, MAPK, regulation of actin cytoskeleton and Rap1 signaling pathway. **(G)** KEGG analysis of differentially expressed lncRNA co-expressed with mRNA in ADNS versus ADF. Most genes focus on the pathway of phagosome, synaptic vesicle cycle and adipocytokine signaling pathway (KEGG pathways: https://www.kegg.jp/kegg/kegg1.html).

To identify the functions of lncRNAs, the upstream and downstream genes of lncRNAs were analyzed. We observed 14,906 lncRNA transcripts, including 4311 annotated lncRNAs and 10,595 novel lncRNAs. The lncRNA transcripts corresponded to 4,311 annotated lncRNA genes and 10,595 novel lncRNA genes (Supplementary Table 5). Overall, 46 differentially expressed lncRNAs between WT versus ADNS, including 24 down-regulated and 22 up-regulated, and 68 differentially expressed lncRNAs between ADNS versus ADF, including 39 down-regulated and 29 up-regulated were observed. Additionally, 5 lncRNAs in the intersection of ADNS versus ADF and WT versus ADNS were detected (Fig. 3A,B,C; Supplementary Table 5).

Differentially expressed lncRNA co-located with mRNA in the KEGG enrichment indicates that majority of genes focused on the PI3K-AKT signal pathway in WT versus ADNS. Renin-angiotensin system (RAS) and endocrine and other factor-regulated calcium reabsorption were significant enriched signaling pathways in WT versus ADNS (*p* < 0.05) (Fig. 3D; Supplementary Table 6). The cytokine-cytokine receptor interaction and cGMP-PKG signaling pathway were dominant in ADF versus ADNS. Biosynthesis of unsaturated fatty acids and the pentose phosphate pathway were significantly enriched signaling pathways in ADF versus ADNS (*p* < 0.05) (Fig. 3E; Supplementary Table 6).

Differentially expressed lncRNA co-expressed with mRNA in KEGG enrichment analysis showed that large portion of genes focused on PI3K-Akt signaling pathway, MAPK signaling pathway, regulation of actin cytoskeleton and Rap1 signaling pathway in WT versus ADNS (Fig. 3F; Supplementary Table 6). There were enriched signaling pathways, such as styrene degradation, sesquiterpenoid and triterpenoid biosynthesis, phenylpropanoid biosynthesis in WT versus ADNS. Peroxiredoxin 6 (PRDX6) attenuates Aβ pathology in phenylpropanoid biosynthesis pathway^32^. Our results showed LNC_000304, which in ADNS mice was significantly higher than WT, co-expressed with mRNA of Peroxiredoxin 6 (Prdx6, ENSMUST00000051925).

In ADNS versus ADF, most of genes were involved in the phagosome, synaptic vesicle cycle and adipocytokine signaling pathways (Fig. 3G; Supplementary Table 6). Also, nitrotoluene degradation and phenylpropanoid biosynthesis are enriched signaling pathways in ADNS versus ADF. Our results demonstrated that LNC_002258, which was significantly lower in ADF than ADNS, was co-expressed with mRNA (Novel_000141) in the phenylpropanoid biosynthesis pathway.

### Expression profiles of miRNAs in hippocampal tissues

**Figure 4:**
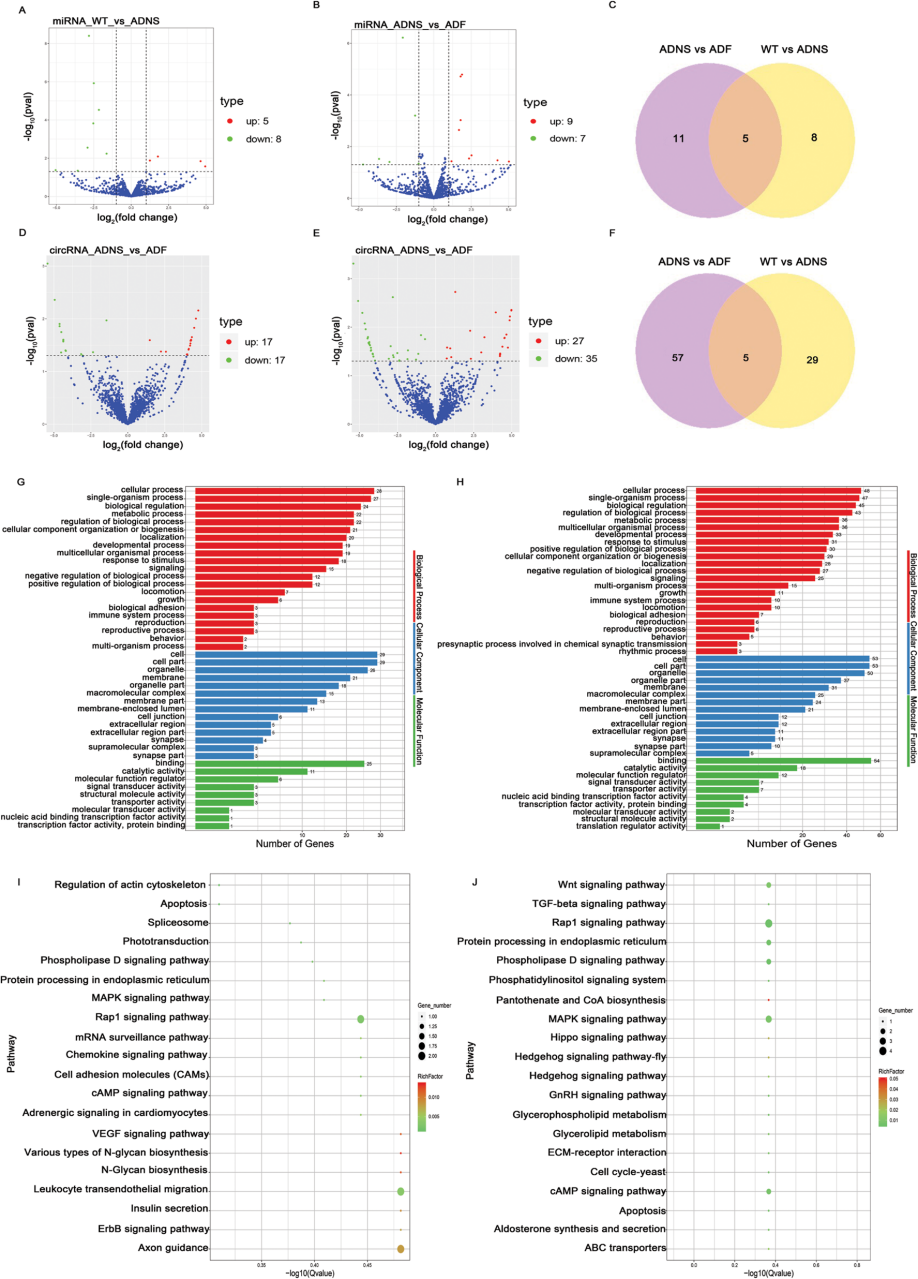
Differentially expressed miRNAs and circRNAs in hippocampal tissues from mice in each group. (**A**) The number of upregulated (5) and downregulated (8) miRNAs between ADNS and WT are shown. **(B)** The number of upregulated (9) and downregulated (7) differentially expressed miRNAs between ADF and ADNS are shown. **(C)** Venn showed 5 miRNAs at the intersection of ADNS versus ADF and WT versus ADNS. **(D)** The number of upregulated (17) and downregulated (17) circRNAs between ADNS and WT are shown. **(E)** The number of upregulated (27) and downregulated (35) differentially expressed circRNAs between ADF and ADNS are shown. **(F)** Venn showed 5 circRNAs at the intersection of ADNS versus ADF and WT versus ADNS. **(G)** GO analysis of 34 differentially expressed circRNAs in WT versus ADNS. The mRNAs were significantly enriched in 521 GO terms (*p* < 0.05). **(H)** GO analysis of the 62 differentially expressed circRNAs in ADNS versus ADF. The mRNAs were significantly enriched in 1226 GO terms (*p* < 0.05). **(I)** KEGG analysis of differentially expressed circRNAs in WT versus ADNS. KEGG analysis of differential cirRNA expression found in WT versus ADNS were involved in signal transduction, including the VEGF signalling pathway, ErbB signalling pathway, MAPK signalling pathway, et al.; they also were involved in the immune system, including Chemokine signalling pathway and Leukocyte transendothelial migration. **(J)** KEGG analysis of differential cirRNA expression found in ADNS versus ADF were involved in signal transduction, including the MAPK signalling pathway, Phosphatidylinositol signalling system, Phospholipase D signalling pathway, Rap1 signalling pathway, TGF-beta signalling pathway, and Wnt signalling pathway (KEGG pathways: https://www.kegg.jp/kegg/kegg1.html).

We first determined whether the miRNA profile of ADNS and ADF was distinct as compared with WT by analysis of differentially expressed miRNAs. We observed 13 significantly and differentially expressed miRNAs, including 5 up-regulated (38.5%) and 8 down-regulated (61.5%) in ADNS versus WT (Fig. 4A; Supplementary Table 7). There were 16 significantly and differentially expressed miRNAs, including 9 up-regulated (56.3%) and 7 down-regulated (43.8%), in ADF as compared with ADNS (Fig. 4B; Supplementary Table 7). There were 5 miRNAs at the intersection of ADNS versus ADF and WT versus ADNS (mmu-miR-141-3p, mmu-miR-183-5p, mmu-miR-182-5p, mmu-miR-96-5p, novel_339) (Fig. 4C; Supplementary Table 7).

### Analysis of circRNA changes in fasudil-treated APPswe/PSEN1dE9 mice

CircRNAs play a vital role in neurodegenerative diseases, particularly in AD^33^. Our results illustrated 34 differentially expressed circRNAs in ADNS versus WT, including 17 up and 17 down (Fig. 4D and Supplementary Table 8). There were 62 differentially expressed circRNAs in ADNS versus ADF, including 27 up and 35 down, (Fig. 4E and Supplementary Table 8). There were 5 at the intersection of ADNS versus ADF and WT versus ADNS, including mmu_circ_0000295 in ADNS and the SRPBM was 168.8, while the SRPBM of ADF and WT was 0. The readouts of mmu_circ_0001551, mmu_circ_0006568 and mmu_circ_0010965, mmu_circ_0011161 were 0 in ADNS, while in WT were around 200 and in ADF returned to WT (Fig. 4F; and Supplementary Table 8).

Next, biological functions of the differentially expressed circRNAs between WT versus ADNS and ADNS versus ADF were identified by gene ontology (GO) and pathway enrichment analysis. Differentially expressed circRNAs between WT versus ADNS were significantly enriched in 521 GO terms (*p* < 0.05), and differentially expressed circRNAs between ADNS versus ADF were significantly enriched in 1226 GO terms (*p* < 0.05) (Supplementary Table 9). Our results showed that the important biological process GO terms were cellular process, single organism process and biological regulation in both WT versus ADNS and ADNS versus ADF. The cellular components involved were cell, cell part, organelle, and membrane; the molecular functions consisted of binding, catalytic activity and the molecular function regulator (Fig. 4G,H and Supplementary Table 9).

KEGG analysis revealed that differential circRNA expression in WT versus ADNS was involved in signal transduction, including the VEGF signaling (ko04370), ErbB signaling (ko04012), and MAPK signaling (ko04010) pathways. The circRNAs were also involved in the immune system, including chemokine signaling (ko04062) and leukocyte transendothelial migration (ko04670). Two genes, including the transient receptor potential cation channel, subfamily C, member 4 (Trpc4, ENSMUSG00000027748: mmu_circ_0007002) and PTK2 protein tyrosine kinase 2 (Ptk2, ENSMUSG00000022607: mmu_circ_0003052), were involved in the axons guidance pathway (ko04360) and, for the latter, also in signal transduction, specifically in the VEGF signaling (ko04370) and ErbB signaling (ko04012) pathways (Fig. 4I and Supplementary Table 9).

However, KEGG analysis of the differential circRNA expression between ADNS and ADF showed the involvement of circRNAs in signal transduction, including the MAPK signaling (ko04010), phosphatidylinositol signaling (ko04070), phospholipase D signaling (ko04072), Rap1 signaling (ko04015), TGF-beta signaling (ko04350), and Wnt signaling (ko04310) pathways (Fig. 4J and Supplementary Table 9). Pantothenate kinase 2 (Pank2, ENSMUSG00000037514: mmu_circ_0005875) was involved in pantothenate and CoA biosynthesis (ko00770). The calcium channel, voltage-dependent, P/Q type, alpha 1A (Cacna1a, ENSMUSG00000034656: mmu_circ_0010666 ) was involved in the synaptic vesicle cycle (ko04721), long-term depression (ko04730), taste transduction (ko04742), MAPK signaling pathway (ko04010), GABAergic synapse (ko04727), cholinergic synapse (ko04725), glutamatergic synapse (ko04724), dopaminergic synapse (ko04728), serotonergic synapse (ko04726), retrograde endocannabinoid signaling (ko04723), and calcium signaling pathway (ko04020) (Supplementary Table 9).

### Construction of the competing endogenous RNA (ceRNA) network

**Figure 5:**
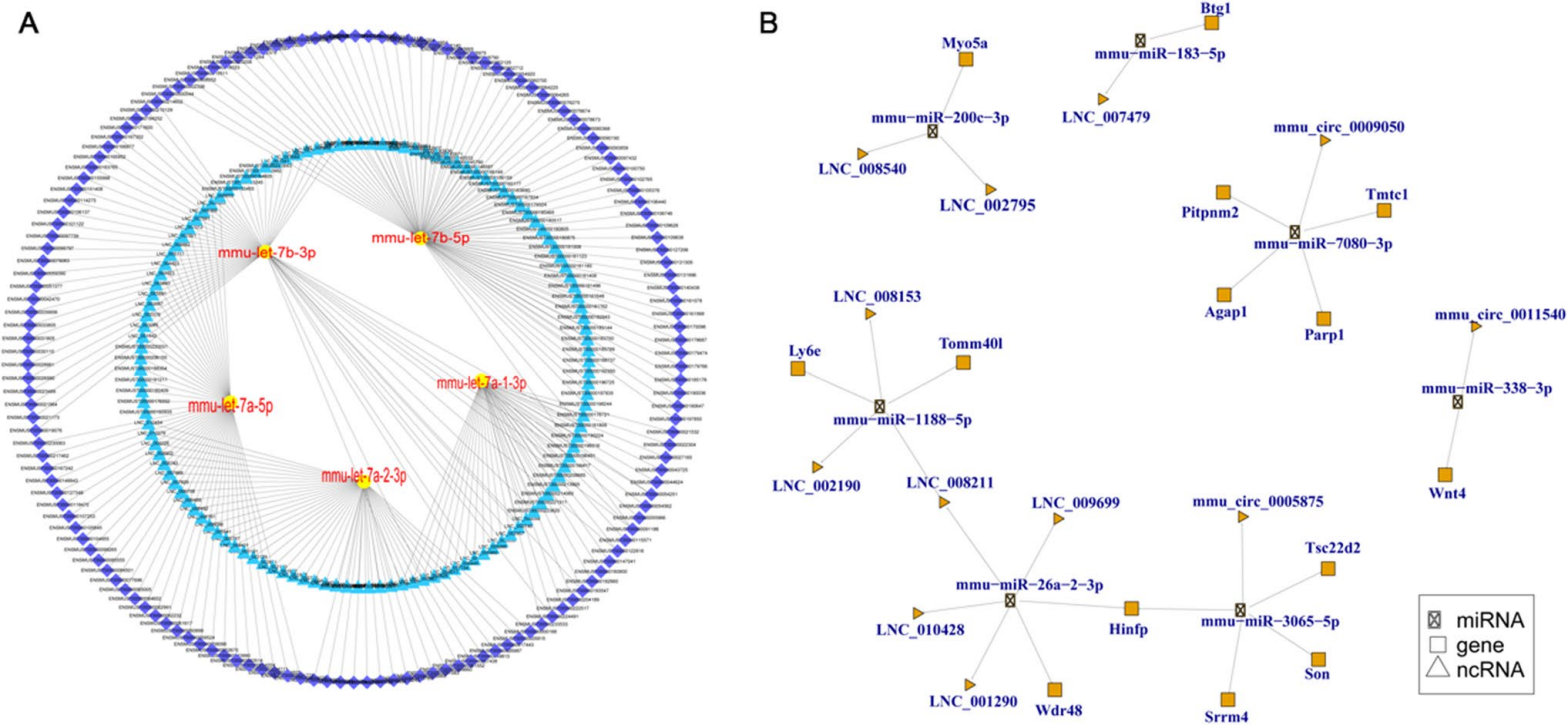
The competing endogenous RNA (ceRNA) network of micRNA, mRNA, lncRNA, and cirRNA. (**A**) The top 300 ceRNA network of whole of miRNA, mRNA, lncRNA, and cirRNA. A yellow ellipse represents miRNA, blue triangle represents lncRNA (unknown: LNC, known: ENSMUST), and purple rhombus represents mRNA. No cirRNA were involved in the top 300. Lines represent correlations. **(B)** CeRNA network of differentially expressed miRNA, mRNA, lncRNA, and cirRNAs. mmu-miR-200c-3p-mediated ceRNA crosstalk between LNC_008540, LNC_002795 and myosin VA (Myo5a, ENSMUST00000123128). mmu-miR-183-5p mediated ceRNA crosstalk between LNC_007479 and BTG anti-proliferation factor 1 (Btg1, ENSMUST00000038377). LNC_008211 is co-expressed with mmu-miR-1188-5p-mediated ceRNA and mmu-miR-26a-2-3p-mediated ceRNA. Histone H4 transcription factor (Hinfp, ENSMUST00000216508) is co-expressed with mmu-miR-26a-2-3p-mediated ceRNA crosstalk and mmu-miR-3065-5p -mediated ceRNA crosstalk.

Construction analysis of the ceRNA network of all the miRNA, mRNA, lncRNA, and circRNA identified the top 300 ceRNA network, was shown in Fig. 5A and Supplementary Table 10. We also analyzed the ceRNA network for differential expression of miRNA, mRNA, lncRNA, and circRNA. The results indicated 7 miRNA-mediated ceRNA crosstalk between 9 lncRNAs, 14 mRNAs and 3 circRNA (Fig. 5B; Supplementary Table 10).

Compared to WT, mmu-miR-200c-3p and mmu-miR-183-5p were significantly lower in ADNS while mmu-miR-7080-3p was significantly higher in ADF; compared to ADF, mmu-miR-183-5p was also significantly lower in ADNS. Compared to ADNS, mmu-miR-338-3p, mmu-miR-1188-5p, mmu-miR-26a-2-3p, and mmu-miR-3065-5p were all significantly lower while LNC_008211 and Histone H4 transcription factor (Hinfp, ENSMUST00000216508) in ADF were significantly higher (Supplementary Tables 2, 5 and 7).

The mmu-miR-200c-3p-mediated ceRNA crosstalk between LNC_008540, LNC_002795 and myosin VA (Myo5a, ENSMUST00000123128) was one of the three myosin V heavy-chain genes, belonging to the myosin gene superfamily. mmu-miR-183-5p was one of the AD-related miRNAs, mediating the ceRNA crosstalk between LNC_007479 and Btg1 (ENSMUST00000038377), which is involved in the PI3K/Akt/VEGF signal pathway. mmu-miR-7080-3p-mediated ceRNAs played an important role in the crosstalk between mmu_circ_0009050 and 4 mRNAs (Fig. 5B; Supplementary Table 10).

The mmu-miR-338-3p-mediated ceRNA cross-talked between mmu_circ_0011540 and Wnt4 (ENSMUST00000045747). The mmu-miR-1188-5p-mediated ceRNA cross-talked between LNC_002190, LNC_008153, LNC_008211, and translocase of outer mitochondrial membrane 40-like (Tomm40l, ENSMUST00000005817), lymphocyte antigen 6 complex, and locus E (Ly6e, ENSMUST00000169343). The mmu-miR-26a-2-3p-mediated ceRNA cross-talked between LNC_001290, LNC_008211, LNC_009699, LNC_010428, WD repeat domain 48 (Wdr48, ENSMUST00000215307), and Hinfp (ENSMUST00000216508). The mmu-miR-3065-5p-mediated ceRNA cross-talked between mmu_circ_0005875 and Hinfp (ENSMUST00000216508), serine/arginine repetitive matrix 4 (Srrm4, ENSMUST00000076124), TSC22 domain family, member 2 (Tsc22d2, ENSMUST00000099090), and Son DNA binding protein (Son, ENSMUST00000117633) (Fig. 5B; Supplementary Table 10).

We also observed that LNC_008211 co-expressed with mmu-miR-1188-5p-mediated ceRNA and mmu-miR-26a-2-3p-mediated ceRNA. Hinfp (ENSMUST00000216508) co-expressed with mmu-miR-26a-2-3p-mediated ceRNA crosstalk and mmu-miR-3065-5p-mediated ceRNA crosstalk (Fig. 5B; Supplementary Table 10).

### Validation of differentially expressed RNAs by real time quantitative PCR (RT-PCR)

**Figure 6:**
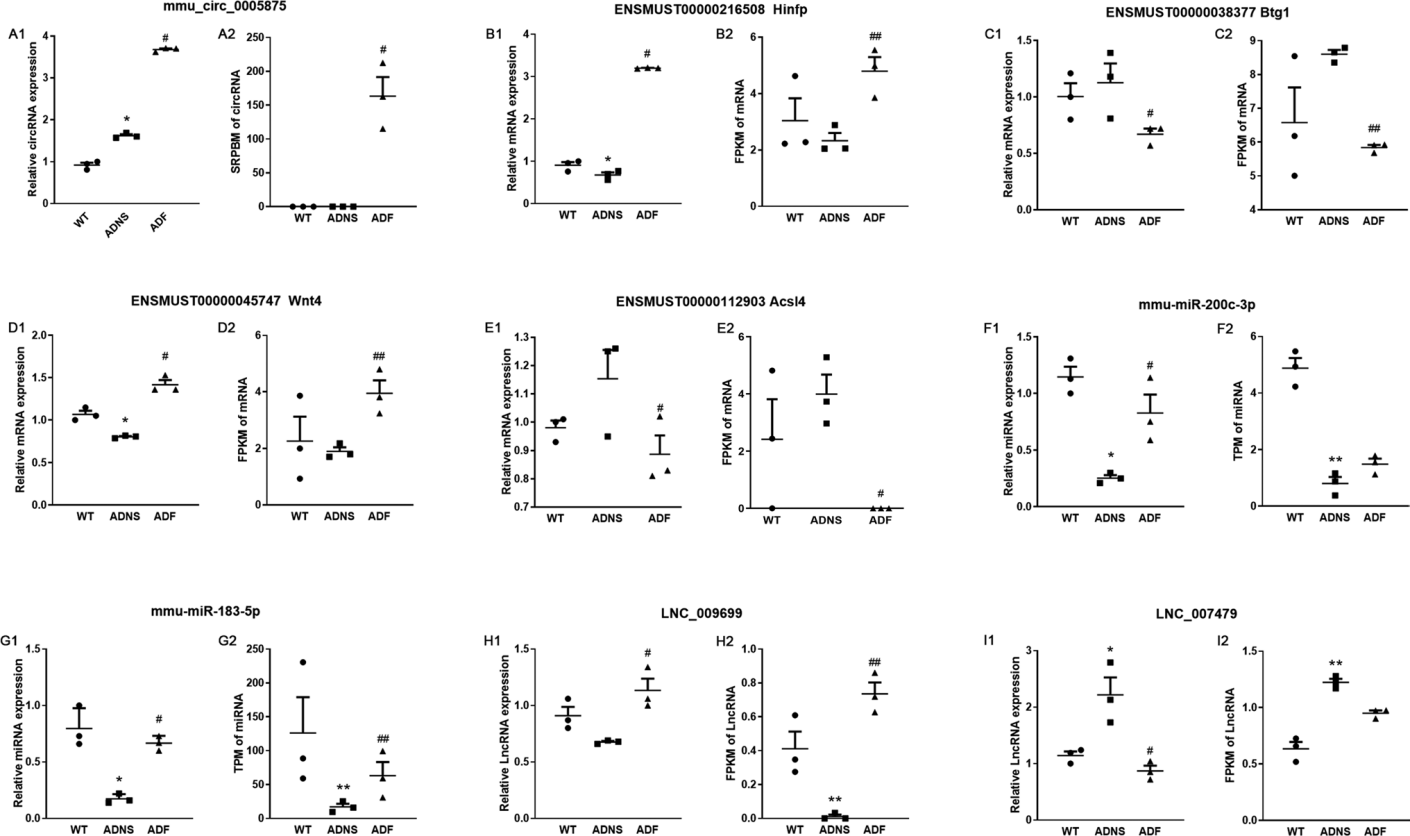
Validation of Differentially Expressed RNAs in hippocampal tissues from mice in each group. (**A**) Expression changes for select circRNA using RT-PCR (left) and high-throughput sequencing results (right) among WT, ADNS, and ADF samples. **(B–E)** Expression changes for select mRNAs using RT-PCR (left) and high-throughput sequencing results (right) among WT, ADNS, and ADF samples. **(F,G)** Expression changes for select miRNA using RT-PCR (left) and high-throughput sequencing results (right) among WT, ADNS, and ADF samples. **(H,I)** Expression changes for select lncRNAs using RT-PCR (left) and high-throughput sequencing results (right) among WT, ADNS, and ADF samples. *p < 0.05, **p < 0.01 versus WT; #*p* < 0.05, ##*p* < 0.01 versus ADNS. The Dunnett’s test were used for statistical analysis.

To verify the specific differences in the abundance of certain RNAs in ADNS, WT and ADF, we selected LNC_009699, LNC_007479, mmu-miR-200c-3p, mmu-miR-183-5p, mmu_circ_0005875, Hinfp (ENSMUST00000216508), Btg1 (ENSMUST00000038377), Wnt4 (ENSMUST00000045747), and Acsl4 (ENSMUST00000112903) mRNAs from the constructed network to perform validation by RT-PCR. RT-PCR expression patterns of the 1 circRNA, 2 lncRNAs, 2 miRNAs, and 4 mRNAs were similar to the high-throughput sequencing results and verified the reliability of sequencing results (Figs. 6; Supplementary Table 11).

Compared to ADNS, mmu_circ_0005875 and Hinfp (ENSMUST00000216508) were both significantly higher in ADF (Fig. 6A1,A2,B1,B2). Compared to WT, Wnt4 (ENSMUST00000045747) was significantly or tended to be lower in ADNS, which was reversed in ADF (Fig. 6D1,D2). Expression of Acsl4 (ENSMUST00000112903) was higher in ADNS, but were significantly reduced in ADF (Fig. 6E1,E2). Compared to WT, mmu-miR-200c-3p was significantly lower in ADNS, which was prevented in ADF (Fig. 6F1,F2). A similar case was observed for LNC_009699 (Fig. 6H1,H2). These data were consistent with the results obtained by the transcriptome sequencing and implied RNAs regulatory roles in AD development and fasudil treatment. We performed the RT-PCR in order to explore the function of LNC_007479 and mmu-miR-183-5p regulation of the expression of Btg1. Btg1 tended to be increased in ADNS relative to WT, but was significantly reduced in ADF (Fig. 6C1,C2). Compared to WT, mmu-miR-183-5p was significantly lower while LNC_007479 higher in ADNS; all of which were reversed in ADF (Fig. 6G1,G2,I1,I2), suggesting that LNC_007479 is negatively related to mmu-miR-183-5p and positively to Btg1.

## Discussion

Fasudil is the most commonly used Rho kinase inhibitor, as well as an effective calcium channel antagonist and vasodilator. It crosses the blood brain barrier and clinically used in the treatment of cerebral artery spasm after subarachnoid haemorrhage^34,35^. Our previous published study showed that fasudil improve cognitive function in Morris water maze test and further we used same batches of mice, where feces were used for gut microbiota and metabolites, and hippocampus for transcriptome study^36^. APPswe/PSEN1dE9 mice treated with fasudil showed significant decreased in latency to locate the platform, latency of first entrance into target zone and increased time spent in target zone. Morris water maze test suggested the reversal of learning and memory impairment of AD mice^36^. Fasudil retains the BBB integrity by up-regulating expression of tight junction proteins ZO-1 and occludin has been reported by our studies^37–39^. Activation of ROCK in astrocytes causes retraction and affects the BBB, where ROCK-2 is predominant isoform and acts as the key factor in the function and maintenance of BBB^39^. Our previous studies have revealed the potent effect of fasudil in the CNS, preventing BBB leakage and Aβ deposition^40^. We also reported protective effect of fasudil by clearing Aβ or p-Tau in primary neurons^41^. In this study, fasudil treatment ameliorated Aβ and p-Tau in the hippocampus of APPswe/PSEN1dE9 mice and transcriptome changes in AD mice were similar to vehicle treatment control. We also observed that protein-coding genes, miRNAs, circRNA, and lncRNA transcripts regulates the pathogenesis of AD. Recent studies have shown that lncRNAs, circRNAs, miRNAs, and mRNAs form large-scale ceRNA crosstalk networks, which have exciting implications in gene regulation at the transcriptional level during physiological and pathophysiological processes^42–46^. Although lncRNAs and circRNAs have been identified and reported in several diseases, however, their functions remain largely unknown^47,48^. We performed a full transcriptome analysis of lncRNAs, circRNAs, miRNAs, and mRNAs by using high-throughput sequencing to identify their functions.

DEMs in ADNS versus ADF were enriched in glycosaminoglycan biosynthesis-keratan sulfate (KS) pathways. KS is an extracellular glycan in the brain and plays an important role in AD and neuritis plaque formation^49^. The maturation of N-glycan requires modification of GlcNAc by catalysis of Fucosyltransferase 8 (Fut8) and the action of Beta-1,4-Galactosyltransferase 4 (B4galt4). GlcNAc6ST1 regulates microglial functions in Alzheimer’s pathology^50^. The increased Fut8 expression during aging could make the IGF-1 signaling pathway more sensitive in an older organism^51^. Our results showed that B4galt4 (ENSMUST00000023482) was significantly higher in ADF compared to ADNS, suggesting that fasudil enhances the regulation of B4galt4 in the maturation process of N-glycan. In addition, the Fut8 transcript (ENSMUST00000062804) was significantly lower in ADF compared to ADNS (− 0.8-fold); in contrast, the other Fut8 transcript (ENSMUST00000171770) was significantly higher in ADF compared to ADNS (11.6-fold). FUT8 changed differentially, which is inconsistent with previous studies^51^. This may be related to polymorphism, but needs further verification.

DEMs between ADNS-ADF were enriched in Quorum sensing pathways. Acsl4 (Ensmust00000112903) was found to be significantly increased in subarachnoid hemorrhage (SAH). Inhibition of Acsl4 expression reduces inflammatory responses, blood–brain barrier (BBB) damage, oxidative stress, and increases the number of surviving neurons after SAH^52^. Acsl4 significantly increases protein damage, lipid peroxidation markers and related signaling molecules in hearts from AD mice^53^. Our results showed significantly low Acsl4 (ENSMUST00000112903) in ADF as compared to ADNS, suggesting that fasudil alleviates the impairment caused by Acsl4 in AD mice. The transcriptome sequencing quantitative results are consistent with RT-PCR. It is worth discussing as candidate biomarkers that are positively altered in AD mice after treatment.

MicroRNAs are important post-transcriptional regulators of gene expression and implicated in numerous biological and pathological processes including neurodegenerative diseases^54^. There are 5 miRNAs at the intersection of ADNS versus ADF and WT versus ADNS, including mmu-miR-141-3p, mmu-miR-183-5p, mmu-miR-182-5p, mmu-miR-96-5p, and novel_339. Our results showed that mmu-miR-183-5p in ADNS was significantly lower than that in WT and reversed in ADF. Several miRNAs are also decreased in the abundance in some diseases, which include 182 cluster such as miR-182-5p, miR-183-5p, and mmu-miR-200c-3p^55^.

We investigated the function of the lncRNAs and circRNAs in response to fasudil treatment in AD mice. Our results demonstrated that circRNAs were differentially expressed in the hippocampus, including 17 up and 17 down in ADNS versus WT, and 27 up and 35 down in ADNS versus ADF. The recent identification of several circRNAs as vital regulators of miRNAs underlines the increasing complexity of ncRNA-mediated regulatory networks^56^. LncRNAs are mainly involved in epigenetic modifications that regulate gene expression such as DNA methylation, histone acetylation, and transcriptional activation, with positive regulatory effects on target genes^44,57^. A gene pair-based method incorporated with lncRNA-mediated gene regulatory networks has been established to identify module biomarkers associated with different brain regions and comprehensively develop biomarker prediction methods and therapeutic strategies for the treatment of AD patients^58^. We demonstrated that protein-coding genes, miRNAs, circRNA, and lncRNA transcripts are candidate regulators for development of AD. Further studies are needed to verify this.

We also constructed a ceRNA network by integrating competing relationships between protein-coding transcript-miRNA and lncRNA transcript-miRNA pairs. Btg1 (ENSMUST00000038377), a member of the cell cycle inhibitory gene family^59^, can inhibit cell proliferation, promote cell apoptosis, and regulate cell cycle progression and differentiation^60–62^. Btg1 is expressed in the developing and adult brain^63^, but its function in neural tissues is unclear. Our results demonstrated that the high level of Btg1 in ADNS was significantly reduced in ADF. Similarly, the high level of LNC_007479 in ADNS, which is positively correlated with Btg1, was significantly reduced in ADF. mmu-miR-183-5p had decreased expression in ADNS than WT and ADF and was negatively correlated with Btg1. There is a competitively inhibitory effect between LNC_007479 and mmu-miR-183-5p. More specifically, mmu-miR-183-5p negatively regulates the Btg1 mRNA; LNC_007479 binding to mmu-miR-183-5p weakens the inhibitive effect of the miRNA. Upregulation of miR-183-5p along with the downregulation of miR-206-3p and miR-381-3p may serve as putative biomarkers of 4,4′-methylene diphenyl diisocyanate (MDI) exposure^58^. Several miRNAs, including mmu-miR-183-5p, are significantly decreased in a few diseases. Our results demonstrated that while few genes could be involved in the PI3K/Akt pathway, Btg1 appears to be one of these genes. Dysregulation of the PI3K/Akt pathway is implicated in a number of human diseases including cancer, diabetes, cardiovascular disease, and neurological diseases. Our previous results demonstrated that fasudil reversed Aβ_1-42_-induced apoptosis that could be related to Btg1^41^. Therefore, we believe that LNC_007479, miR-183-5p and Btg1 could be potential candidate biomarkers which are positively altered in AD mice, and reverse after fasudil treatment.

Hinfp is the only known transcription factor required for histone H4 gene expression, which binds directly to a unique H4 promoter-specific element to regulate histone H4 transcription at the G1/S phase transition^64,65^. mmu-miR-3065-5p-mediated ceRNAs crosstalk between mmu_circ_0005875 and Hinfp (ENSMUST00000216508). Our sequence result analysis showed that mmu_circ_0005875 of ADF was significantly higher compared to ADNS, and Hinfp in ADF was significantly higher than that in ADNS, and RT-PCR validated it same, suggesting a positive relationship among them. In addition, mmu_circ_0005875 was positively correlated with Hinfp*.* Therefore, Hinfp could be a candidate biomarker that is positively altered in AD mice after treatment.

Wnt signaling pathway could be a potential target for the treatment of AD due to its close association in pathogenesis of AD. Loss of Wnt signaling enhance neuronal susceptiblity to Aβ—induced apoptosis, while activation of Wnt signaling prevent Aβ induced neuronal death and behavioural deficit^66,67^. Our results showed Wnt4 (ENSMUST00000045747) mRNAs in ADF are significantly higher than in ADNS. A recent study in mouse model of AD reported that dickkOPF-1 (DKK-1), an inhibitor of a typical Wnt signaling pathway, is induced by Aβ and promotes the atypical Wnt-PCP pathway^68^. Fasudil antagonize the Wnt-PCP pathway, restored balance between typical and atypical Wnt signaling pathways and reduces Aβ dependent synaptic toxicity^69^. Regulation of Wnt signaling pathways in central nervous system is expected as powerful tools to control neural immune homeostasis. We also observed mmu-miR-200c-3p is ADNS significantly lower than WT, higher expression of LNC_009699 in ADF and WT as compared to ADNS. There is a probable competitive inhibitory effect between LNC_009699 and mmu-miR-200c-3p; mmu-miR-200c-3p negatively regulates Wnt4 (ENSMUST00000045747) mRNAs expression. These candidate biomarkers are positively altered in AD mice after treatment.

In conclusions, we discovered mRNAs, miRNAs, circRNAs and lncRNAs transcripts that are candidate regulators of development of AD. We also constructed a ceRNA network by integrating competing relationships between protein-coding transcript-miRNA and lncRNA transcript-miRNA pairs. Further, we identified several lncRNA transcripts predicted to regulate the Acsl4, Btg1, Hinfp and Wnt4. The RT-PCR expression patterns were similar to high-throughput sequencing results, which verified the reliability of sequencing results. These four genes (ENSMUST00000112903, ENSMUST00000038377, ENSMUST00000216508 and ENSMUST00000045747) could be potential biomarkers of AD and the targets of fasudil treatment.

## Materials and methods

### Animals and treatment

Animal Ethics Committee of Shanxi Datong University approved all procedures. All the experiments were performed in compliance with the guidelines and regulations of the Administration Office of the International Council for Laboratory Animal Science and ARRIVE guidelines. Male APPswe/PSEN1dE9 mice and the C57BL/6 background (8 months old) were obtained from Beijing Huafukang Bioscience CO., LTD (HFK, Beijing, China). Mice were placed in the pathogen free facilities at the Institute of Brain Science, Shanxi Datong University, pen), separated in a temperature and humidity controlled room with 12-h light/12-h dark cycle. Animals had ad libitum access to food and water. The three groups of mice were selected: (1) vehicle-treated mice (ADNS) were administered normal saline (volume was adjusted similar to fasudil treatment); (2) fasudil (ADF) mice were injected daily with fasudil (Tianjin Chase Sun Pharmaceutical Co., Ltd.), 25 mg/kg/day, i.p. for 16 weeks; (3) age- and gender-matched C57BL/6 (WT) mice (8 months) were injected with the same volume of normal saline (n = 6 per group). 9 mice (n = 3) were sacrificed by cervical dislocation at the end of treatment, and the hippocampal tissues were rapidly collected, fixed with 4% cold paraformaldehyde and embedded in paraffin for immunofluorescence staining. Another, 9 mice (n = 3, biological replicate) were sacrificed at the end of treatment, and snap-frozen in liquid nitrogen and stored at − 80 °C for RNA extraction and RNA-sequencing.

### Immunofluorescence staining for Aβ plaques and p-Tau

The hippocampal sections were permeabilized with 0.1% Triton X-100 for 15 min, and blocked with 1% bovine serum albumin (BSA) for 30 min at room temperature (RT). Sections were then incubated with anti-Tau (phospho-S404) (ab92676, mouse monoclonal primary antibodies, dilution of 1:200, Abcam, USA), anti-Aβ1-42 (GB111197, rabbit monoclonal primary antibodies, 1:500, Servicebio, China) at 4ºC overnight. Slides were incubated with FITC or Cy3-conjugated secondary antibodies (Invitrogen, USA) for 1 h at RT. The modified coverslips were observed with a confocal microscopy (Olympus FV1000, Japan). Images were taken at 10× (200 μm) and 60× (20 μm).

### RNA extraction and sequencing

The hippocampal samples were extracted using Trizol reagent (Thermo Fisher) (n = 3 mice per group). Purity and concentration was determined using the NanoPhotometer® spectrophotometer (IMPLEN, CA, USA). The sample quality was assessed using Agilent Bioanalyzer 2100 and sequencing libraries were prepared using the concentration of total RNA (3 μg). Directional RNA Library Prep Kit for Illumina® (NEB, USA) following the manufacturer’s recommended protocol. The samples was performed on a cBot Cluster Generation System using TruSeq PE Cluster Kit v3-cBot-HS (Illumia).

### mRNA and lncRNA-Seq analysis

Raw data in fastq format was processed first by removing potential rRNA contamination by mapping to known rRNA gene sequences. Next, an in-house tool was used to remove low quality read pairs, which includes reads with adapter sequences, reads with consequtive Q value < 20 at the ends, and reads less than 50 bp. QC of the sequencing data was assessed using FastQC. Hisat2 was used to map reads to the reference genome. Reference genome and gene model annotation files were downloaded from the genome website directly^70^. The mapped reads of each sample were assembled and convert to FPKM by StringTie (v1.3.1). We used three tools (CNCI, CPC2 and PLEK) to predict coding potential of each transcript. Transcripts predicted without coding potential were selected as candidate lncRNAs and the remaining transcripts were deemed as mRNA. Transcripts not overlapping with known gene annotations were considered as “novel transcripts”. The Ballgown suite includes functions for interactive exploration of the transcriptome assembly, visualization of transcript structures and feature-specific abundance for each locus and post hoc annotation of assembled features to annotated features^71^. DESeq2 was used to analyse differential gene expression for significance analysis (*p* < 0.05 after Benjamin-Hochberg correction)^72^. Cuffdiff provides statistical routines for determining differential expression in digital transcript or gene expression data using a model based on the negative binomial distribution^73^. We searched co-located (the screening range was less than 100 K between lncRNA and mRNA) and co-expressed (the correlation coefficient between lncRNA and mRNA was greater than 0.95) target genes of lncRNAs, and analyzed their functional annotations. Gene Ontology (GO)^74^ and KEGG^75^ enrichment analysis of differentially expressed genes or lncRNA target genes were implemented by the GOseq R package and KOBAS software (http://www.genome.jp/kegg/). The terms with corrected p-values less than 0.05 were considered significantly enriched by differentially expressed genes^75–77^.

### circRNA-Seq analysis

circRNA-seq analysis was performed by combining the results of two software, find-circ and CIRI2 to reduce false positives^78–80^. Only the results predicted by both tools were used for subsequent analyses. Quantification was carried out using SRPBM (Spliced Reads per Billion Mapping)^81^. Differential circRNA expression was carried out using DESeq2 (*p* < 0.05). Functional annotation of circRNA was based on GO and KEGG annotation. Enrichment analysis was performed as described above in the methodology.

### miRNA-Seq analysis

The miRNA library was constructed using NEBNext® Multiplex Small RNA Library Prep Set for Illumina® (NEB, USA). Index codes were added to attribute sequences for each sample as per the manufacturer’s recommendations. Briefly, the cDNA library size was separated using PAGE gel and small RNA between 18 and 40 bp was excised and purified. Bowtie2 was used to compare miRNAs to the reference genome, and the distribution of miRNAs were analyzed^82^.

### Construction of competing endogenous RNA (ceRNA) network

miRNAs, the target mRNAs, lncRNAs, and circRNAs with Pearson’s correlation coefficient < − 0.6 & *p* < 0.05 were selected for analysis. Based on the data of differentially expressed protein-coding transcripts, miRNA, and lncRNA transcripts, we used Miranda (v3.3a) to identify the biological targets of each miRNA from the protein-coding and lncRNA transcripts that showed a significant negative correlation with miRNA expression^83^. We subsequently obtained the protein-coding transcript-miRNA and lncRNA transcript-miRNA pairs and further constructed the competing endogenous RNA (ceRNA) network^78^.

### Quantitative real-time quantitative PCR

To confirm the transcriptomic analysis results, circ_0005875, Hinfp, Btg1, Wnt4, Acsl4, miR-200c-3p, miR-183-5p, LNC_009699, LNC_007479 were subjected to real-time PCR. Total RNA was isolated using the RNA Extraction Kit (Servicebio, China), and cDNA was quantified by RT-PCR using ServicebioRT First Strand cDNA Synthesis Kit (Servicebio, China). The fold change in gene expression was calculated using the 2^-ΔΔCt^ method with the house keeping genes, U6 as the miRNA internal control and GAPDH as the internal control of mRNA, lncRNA, and circRNA. The primer sequences we used are shown in Table 1.

**Table 1:** Primer sequences used in the experiment.

Primer	sequence
circ_0005875-F	AAGAGAGAGGCGGCCAGTAA
circ_0005875-R	AAGGCCATCAAGGAAGAAAGG
Hinfp-F	CCAGTCTCCGAAACCCTCAA
Hinfp-R	TAAAGGCATCCAAGAACAAAGG
Btg1-F	GCCACCATGATAGGCGAGAT
Btg1-R	ATCCGGTAGGACACTTCGTAGG
Wnt4-F	GGCTCCTGCGAGGTAAAGAC
Wnt4-R	GATGTCCTGCTCACAGAAGTCC
Acsl4-F	CTTGAGCGTTCCTCCAAGTAGAC
Acsl4-R	AGCACATGAGCCAAAGGTAAGT
miR-200c-3p-F	CTCAACTGGTGTCGTGGAGTCGGCAATTCAGTTGAGTCCATCAT
miR-200c-3p-R	ACACTCCAGCTGGGTAATACTGCCGGGTAAT
miR-183-5p-F	CTCAACTGGTGTCGTGGAGTCGGCAATTCAGTTGAGATTGAATT
miR-183-5p-R	ACACTCCAGCTGGGTATGGCACTGGTAGAA
LNC_009699-F	AAGGCATTATAGGCTTGAACGG
LNC_009699-R	ATACCTTGAAGACAACAGTGGCC
LNC_007479-F	CGTAGTCCGTAGGAGGTCGATC
LNC_007479-R	GAAGAAGAAGTATGTGACGCTGTGT
U6-F	CTCGCTTCGGCAGCACA
U6-R	AACGCTTCACGAATTTGCGT
GAPDH-F	CCTCGTCCCGTAGACAAAATG
GAPDH-R	TGAGGTCAATGAAGGGGTCGT

The forward and reverse primer of circ_0005875, Hinfp, Btg1, Wnt4, Acsl4, miR-200c-3p, miR-183-5p, LNC_009699, LNC_007479 were designed.

### Statistical analysis

SPSS software (International Business Machines Corporation, IBM, USA) was used for statistical analysis. All data was expressed as means ± SEM. Dunnett’s test was used to compare two groups, using ADNS as a control, WT and ADF were compared with ADNS. We used Dunnett's post hoc test for analysis of immunofluorescent intensity quantification and quantitative RT-PCR analysis. A value of *p* < 0.05 was considered statistically significant.

## Supplementary Information

Supplementary Table 1.

Supplementary Table 2.

Supplementary Table 3.

Supplementary Table 4.

Supplementary Table 5.

Supplementary Table 6.

Supplementary Table 7.

Supplementary Table 8.

Supplementary Table 9.

Supplementary Table 10.

Supplementary Table 11.

Supplementary Legends.
