# Supplementary material for: Transcriptome analysis of fasudil treatment in the APPswe/PSEN1dE9 transgenic (APP/PS1) mice model of Alzheimer’s disease
Source: Sci Rep. 2022 Apr 22;12:6625. doi: 10.1038/s41598-022-10554-9 (PMC9033779; doi:10.1038/s41598-022-10554-9)
Supplement: Supplementary file 12 — Supplementary Legends. [file 41598_2022_10554_MOESM12_ESM.docx]

**Supplementary data availability**

Supplementary data is available at following link

Digital Object Identifier: 10.6084/m9.figshare.17069540

https://figshare.com/s/9967efb0003d3c78c2de/

**List of supplementary tables legends**

**Supplement table 1**: Quality of sequencing data in fasudil-treated AD mice

**Supplement table 2**: Differentially expressed mRNA in fasudil-treated AD mice

**Supplement table 3:** GO annotations of differentially expressed mRNAs in fasudil-treated AD mice

**Supplement table 4:** KEGG pathways of differentially expressed mRNAs in fasudil-treated AD mice

**Supplement table 5**: Differentially expressed lncRNA in fasudil-treated AD mice

**Supplement table 6**: Differentially expressed lncRNA co-located and co-expressed with mRNA in the KEGG enrichment in fasudil-treated AD mice

**Supplement table 7:** Differentially expressed miRNA in fasudil-treated AD mice

**Supplement table 8**: Differentially expressed circRNA in fasudil-treated AD mice

**Supplement table 9**: GO annotations and KEGG pathways of differentially expressed circRNAs in fasudil-treated AD mice

**Supplement table 10:** ceRNA network of all the miRNA, mRNA, lncRNA, and circRNA; ceRNA network for differential expression of miRNA, mRNA, lncRNA, and circRNA

**Supplement table 11:** Validation of differentially expressed RNAs by real time quantitative PCR in fasudil-treated AD mice
